# Crosstalk Between the Oncoproteins of High‐Risk Human Papillomaviruses Types 16 and 18 in Colorectal Cancer Cell Models

**DOI:** 10.1002/cnr2.70197

**Published:** 2025-06-04

**Authors:** Queenie Fernandes, Varghese Philipose Inchakalody, Sarra Mestiri, Takwa Bedhiafi, Shereena Hydrose, Sara S. Bashraheel, Maysaloun Merhi, Said Dermime, Ala‐Eddin Al Moustafa

**Affiliations:** ^1^ College of Medicine, QU Health, Qatar University Doha Qatar; ^2^ Translational Cancer Research Facility National Center for Cancer Care and Research, Hamad Medical Corporation Doha Qatar; ^3^ Qatar Biomedical Research Institute (QBRI), Hamad Bin Khalifa University, Qatar Foundation Doha Qatar; ^4^ Biomedical Research Center, QU Health, Qatar University Doha Qatar; ^5^ Oncology Department McGill University Montreal Quebec Canada

**Keywords:** colorectal cancer, epithelial‐mesenchymal transition, human papillomavirus, oncoproteins, viral co‐infection

## Abstract

**Background:**

Colorectal cancer (CRC) represents a major fraction of the total cancer burden worldwide. It has been recently identified that various high‐risk Human Papillomaviruses (HPVs) are present in human CRCs, where they play a critical role in the development and progression of the cancer.

**Aims:**

In this study, we explored the synergistic effect of the E6/E7 viral oncoproteins of the two most frequently observed HPV types (16 and 18) on KRAS and TP53 mutant CRC cell models.

**Methods:**

We performed an experimental in vitro study utilizing lipofection to transfect KRAS and TP53 mutant CRC cell models (HCT 116 and HT‐29 respectively) with E6/E7 oncoproteins of HPV types 16 and 18 individually and in combination. Subsequently, we assessed their synergistic effect on cell proliferation, invasion, migration, and survival. In addition, we also compared the protein expression patterns of key epithelial‐mesenchymal transition (EMT) biomarkers like E‐cadherin, fascin, and vimentin among transfected, co‐transfected, and wild‐type cells.

**Results:**

We found that the co‐expression of E6/E7 of HPV types 16 and 18 enhanced cell proliferation, invasion, migration, and survival in both cell models. Interestingly, this was also accompanied by the deregulation of all three EMT biomarkers, E‐cadherin, fascin, and vimentin. The synergistic effect of the viral oncoproteins in promoting cancer was more pronounced in TP53 mutant cells (HT‐29) as compared to KRAS mutant cells (HCT 116). We also report that HPV type 18 can induce a greater and more sustained oncogenic outcome as compared to HPV type 16.

**Conclusion:**

Our data indicate that co‐expression of the E6/E7 oncoproteins of HPV types 16 and 18 can enhance oncogenic processes in CRC, especially TP53 mutant CRC.

## 1 Introduction

Colorectal cancer (CRC) constitutes a significant portion of the global cancer burden [1]. It is the second most prevalent cancer in women, following breast cancer, and the third most common in men, after prostate and lung cancers [2]. Hence, it emerges as one of the most prevalent types of cancer globally and the second leading cause of cancer deaths, estimated at 9.3 million deaths annually, according to the most recent Global cancer statistics for 2022 [3]. The causes of CRC are generally attributed to gene mutations and lifestyle factors, including smoking [4], alcohol intake [5], obesity [6] as well as bacterial and viral infections [7, 8].

Cancers in general are considered to be a genetic disease. In particular, mutations accumulating in the DNA‐repair genes, oncogenes, and tumor suppressor proteins often lie at the heart of oncogenesis [9].

Nearly 70% of CRC cases are known to arise out of sporadic mutations [10]. The molecular etiology of CRC exhibits notable heterogeneity, with mutations potentially affecting a range of genes [9]. However, the large majority of CRC cases usually follow a pre‐defined sequence of mutational events, commencing with the formation of adenomas and eventually progressing to carcinomas.

The initial oncogenic event in CRC is defined by a mutation targeting the adenomatous polyposis coli (APC) gene, a well‐known tumor‐suppressor gene [11] that provokes the development of benign adenomas or colorectal polyps. A small percentage of polyps further progress to the formation of carcinomas, most commonly due to subsequent mutations in oncogenes like Kirsten rat sarcoma virus (KRAS) or tumor suppressor genes like tumor protein p53 (TP53) and finally the deleted in colorectal cancer (DCC) gene [9]. The genomic landscape of CRC identifies KRAS as the most frequent mutation in CRC (42%–52%) [12, 13] followed by TP53 [14] (43%) and BRAF (up to 15%) [15].

KRAS mutations commonly occur in codons 12, 13, 59, or 61 [16, 17]. However, point mutations arising in codon 12 represent typical KRAS mutations in CRC [18, 19, 20]. Moreover, KRAS mutations are described as an early pathogenic event that is often a requisite for the progression of colorectal adenoma to CRC [21]. Several studies have reported that the presence of KRAS mutations is linked to a worse prognosis in CRC [22, 23].

On the other hand, around 90% of TP53 mutations are missense mutations, where full‐length proteins harboring a single amino acid chain are expressed [24] that impair p53 function (loss‐of‐function). The TP53 gene encodes for a major tumor suppressor protein (p53) that serves as the central cell‐cycle checkpoint; therefore, its loss triggers tumor progression through uncontrolled cellular proliferation [25]. In addition, TP53 mutations present a classic adenoma to carcinoma succession in CRC. On the whole, mutations in the TP53 gene have been linked to a worse prognosis in CRC in terms of reduced drug response, chemoresistance, and poor survival [26, 27, 28, 29, 30, 31, 32].

Additionally, infectious agents as causative factors in the pathogenesis of CRC are becoming increasingly relevant [33]. In particular, certain viruses, also known as oncoviruses, have been implicated in CRC [7, 34]. For example, high‐risk HPVs have been linked to various cancers [35], including the cervix [36, 37], head, and neck [38, 39] as well as CRC [8, 40, 41]. In addition, according to numerous reports, high‐risk HPV infections in CRC are shown to correlate with vascular invasion, lymph node metastasis as well as tumor grade and size [42, 43, 44, 45]. Interestingly, infections with multiple high‐risk HPV types are implied to show a synergistic effect in tumorigenesis as compared to single HPV infections [46, 47, 48].

HPVs are small, double‐stranded DNA viruses that can infect the epithelial linings of both the anogenital and upper respiratory tracts [49]. They are categorized into low‐risk and high‐risk types based on their potential to cause cancer. Low‐risk HPVs are usually responsible for common anogenital warts [50], while high‐risk HPVs are associated with the development and progression of various human cancers [51]. The early oncoproteins of high‐risk HPVs (E5/E6/E7) are known to be involved in inducing epithelial to mesenchymal transition (EMT), a hallmark of cancer progression, in cervical [52, 53, 54, 55] and other gynecological cancers [56]. Key regulatory genes of EMT, such as E‐cadherin [57, 58, 59, 60], fascin [61, 62, 63] and vimentin [60, 64, 65] have been identified in the pathogenesis of cancer and specifically HPV‐associated cancers [66, 67, 68]. However, the implications of the co‐infection of high‐risk HPVs on EMT and cancer progression in CRC are still not well understood. In a recent analysis, we found that the co‐infection of two or more high‐risk HPVs is a strong indicator of advanced‐stage cancer (stage 3 and 4) and significantly exacerbates the prognosis for CRC patients [69]. Thus, to enhance our comprehension of the intricate molecular and cellular processes associated with high‐risk HPV co‐infection, we herein explored the synergistic effect of the E6/E7 oncoproteins of the two most common high‐risk HPVs (HPV16 and HPV18) in CRC cell models of diverse mutational backgrounds (namely KRAS and TP53 mutants). Our data pointed out that E6/E7 oncoproteins of HPV types 16 (HPV16) and HPV type 18 (HPV18) indeed cooperate to enhance cell proliferation, migration, invasion, and survival, which can lead to metastatic tumorigenesis at distal sites occurring via the deregulation of known EMT biomarkers in co‐infected cells.

## 2 Methods

We performed an experimental in vitro study to transfect KRAS and TP53 mutant CRC cell models (HCT 116 and HT‐29 respectively) with E6/E7 oncoproteins of HPV types 16 and 18 individually and in combination. Subsequently, we assessed their synergistic effect on cell proliferation, invasion, migration, and survival. In addition, we also compared the protein expression patterns of EMT biomarkers like E‐cadherin, fascin, and vimentin among transfected, co‐transfected, and wild‐type cells.

### 2.1 Cell Culture

CRC HCT 116 and HT‐29, cervical cancer cell lines C‐33 A (HPV negative), SiHa (expressing HPV16), and HeLa (expressing HPV18) were purchased from ATCC (Rockville, MD, USA) and cultured in DMEM, GlutaMAX (1X) media Gibco medium (Gibco, Life Technologies) supplemented with 10% fetal bovine serum and 1% PenStrep antibiotic (Thermo Fisher Scientific, USA) as per the manufacturer's instructions. All cells were maintained at 37°C and 5% CO_2_ atmosphere. Experiments were performed when cells were ~ 70%–80% confluent.

### 2.2 Transfection and co‐Transfection of CRC Cell Lines With E6/E7 of HPV16 and HPV18

The pLXSN16E6E7 (Plasmid #52394) and pLXSN18E6E7 (Plasmid #53459) vectors [70] transformed into chemically competent Stbl3 ( *Escherichia coli* ) cells were obtained from Addgene, USA, and cultured in Luria broth/agar (ThermoFisher Scientific, USA). Selection was made through antibiotic resistance to ampicillin (100 μg/mL). Maxiprep purification of plasmid DNA representing the bicistronic E6/E7 of HPV16 and HPV18, respectively, was performed and subsequently used to transfect sub‐confluent CRC cell lines HCT 116 and HT‐29, both distinctly and in combination, in order to generate stable transfected and co‐transfected cell lines, respectively. HPV18‐expressing HeLa cells were also transfected with the pLXSN16E6E7 plasmid in order to generate a positive control for cells co‐transfected with E6/E7 of both HPV16 and HPV18. Briefly, cells (2 × 10^5^) were seeded into 24‐well multiplates and transfected using LipofectAMINE 2000 (Invitrogen) under the conditions suggested by the manufacturer. Forty‐eight hours post‐transfection, cells were selected with Geneticin selective antibiotic (G418) (50 mg/mL) from Gibco (ThermoFisher Scientific, USA) at 800 μg/mL and passaged in culture. Non‐transfected cell lines HCT 116 and HT‐29 were used as controls. Nearly 98% of all transfected cells were healthy after G418 treatment. However, the non‐transfected cells did not survive after 3–4 days of treatment with G418. Transfected cells were passaged twice while maintained on G418. All stable cell lines were subsequently maintained in long‐term culture in DMEM medium supplemented with 10% fetal bovine serum and 1% PenStrep antibiotic (Thermo Fisher Scientific, USA).

### 2.3 RNA Extraction and Reverse Transcription‐PCR (RT‐PCR)

Total cellular RNA was isolated and purified using the RNeasy Mini Kit (Qiagen, Valencia, CA, USA) for both control and transfected/co‐transfected cells. For the RT‐PCR, 10 ng of total RNA was reverse transcribed using SuperScriptTM III One‐Step RT‐PCR System with PlatinumTM Taq DNA Polymerase (Thermo Fisher Scientific, USA). Samples were incubated in the Proflex Thermal Cycler (Thermo Fisher Scientific, Waltham, MA, USA) for reverse transcription at 60°C for 30 min, an initial PCR activation step at 94°C for 2 min followed by 40 thermal cycles. Each cycle consisted of annealing at 94°C for 15 s, at 61°C for 30 s, and at 68°C for 1 min. Final annealing was at 68°C for 5 min. Oligonucleotide primers for HPV16 E7 (Forward Primer: 5′‐ATGCATGGAGATACACCTACATTGCAT‐3′ and Reverse Primer: 5′‐GTTTCTGAGAACAGATGGGGCACAC‐3′) and HPV18 E6 (Forward Primer: 5′‐GCTTTGAGGATCCAACACGG‐3′ and Reverse Primer: 5′‐TGCAGCACGAATGGCACTGG‐3′) were used to confirm the transfection of E6/E7 of HPV16 and HPV18 in the transfected cells, as described previously [41, 71]. The RT‐PCR products were examined by electrophoresis on a 1.5% agarose gel.

### 2.4 Cell Proliferation Assay

Five thousand cells were seeded in 100 μL of cell culture medium per well in 96‐well microplates. Plates were incubated at 37°C, 5% CO_2_ for 24, 48, 72, and 96 h time points. At the end of a time point, 10 μL CCK‐8 solution (Dojindo Molecular Technologies Inc.) was added to each well, and the plates were incubated for 30 min at 37°C, 5% CO_2_. Absorbance was measured at optical density (OD) = 450 nm using a microplate spectrophotometer system. The cell culture medium was used as the blank solution to optimize the absorbance values.

### 2.5 Cell Migration and Invasion (CIM) Assay

This assay was performed using the CELLigence Real‐Time Cell Analysis (RTCA) DP (dual purpose) instrument (Agilent) using the CIM plates that are electronically integrated Boyden chambers. Cells wereserum‐starved by maintaining them in a serum‐free medium (SFM) 8–16 h prior to the experiment. One hundred and sixty μl of complete medium was placed in each well of the lower chamber. The upper chamber was then mounted and 50 μL of SFM medium was added to each well. The plates were placed into the RTCA instrument and allowed to equilibrate for 1 h at 37°C and 5% CO_2_. Subsequently, a background reading was recorded for each well. Cells were prepared in SFM at a seeding density of 3 × 10^4^ cells/well and were plated into each well of the upper chamber to make up a total volume of 150 μL per well. CIM plates were kept at room temperature for 30 min under sterile conditions to allow cells to settle evenly onto the bottom of the upper chamber before being reloaded into the RTCA DP instrument. Cell Index (CI) readings were recorded at 10 min intervals until the end of the experiment (until 24 h). As a control, SFM was placed in a single well of the lower chamber, and readings were used as a baseline.

### 2.6 Cell Survival/Colony Formation Assay

The effect of E6/E7 transfection and co‐transfection on cell survival was evaluated through a clonogenic assay according to the protocol of Franken et al. [72] Briefly, a single cell suspension of 1000 HCT 116 and HT‐29 cells was seeded in a 6‐well plate, in 2 mL of DMEM cell culture medium and monitored for 11 days at 37°C and 5% CO_2_. The growth medium was replaced twice a week. On the 11th day, colonies were washed with PBS, fixed with methanol, and stained with crystal violet solution. Excess stain was washed using ultrapure water and colonies were examined microscopically for further analysis using the EVOS FLc Cell Imaging System, Invitrogen (Thermo Fisher Scientific, USA).

### 2.7 Western Blot for Protein Expression Analysis

Total cell lysates from all transfected and non‐transfected controls were collected in the radioimmunoprecipitation assay (RIPA) buffer (Santa Cruz Biotechnology, USA). Cell lysates were incubated on ice for 30 min and centrifuged at 17000 × g (G‐force) or relative centrifugal force (RCF) for 15 min to extract total proteins. Protein concentrations were determined using the Rapid Gold BCA (Bicinchoninic Acid) protein assay kit (Pierce TM, Thermo Scientific, USA). Forty μg of protein was separated by sodium‐dodecyl sulfate polyacrylamide gel electrophoresis (SDS‐PAGE). Transfer to polyvinylidene difluoride (PVDF) membrane was performed, and membranes were probed with various primary antibodies, namely, E‐cadherin (24E10–#96743, Cell Signaling Technology), Fascin (PA5‐21319, Thermo Fisher Scientific, USA), Vimentin (D21H3–#46173, Cell Signaling Technology), Polymerase delta auxiliary protein (PCNA) (13–3900, Thermo Fisher Scientific, USA) and GAPDH (D16H11–#5174, Cell Signaling Technology). Immunoreactive bands were detected using an enhanced chemiluminescence solution (BioRad, USA) and visualized by a ChemiDocTM MP imaging system (BioRad, USA). Images acquired were analyzed using ImageJ software (National Institutes of Health, USA). The intensity of the bands relative to the GAPDH bands was used to determine and compare protein expression among transfected and non‐transfected cell lines.

### 2.8 Statistical Analysis

Statistical tests were carried out by calculating the mean ± Standard Error of Mean of duplicate/triplicate experimental values. The data were compared using a one‐way analysis of variance (ANOVA) with post hoc testing (multiple comparisons using the Dunnett's test). Microsoft Excel (2016) and GraphPad Prism (Version 8.4.3, San Diego, CA, USA) were used to compute data and perform statistical testing. *p*‐values where *p* < 0.05 were considered significant.

## 3 Results

**FIGURE 1:**
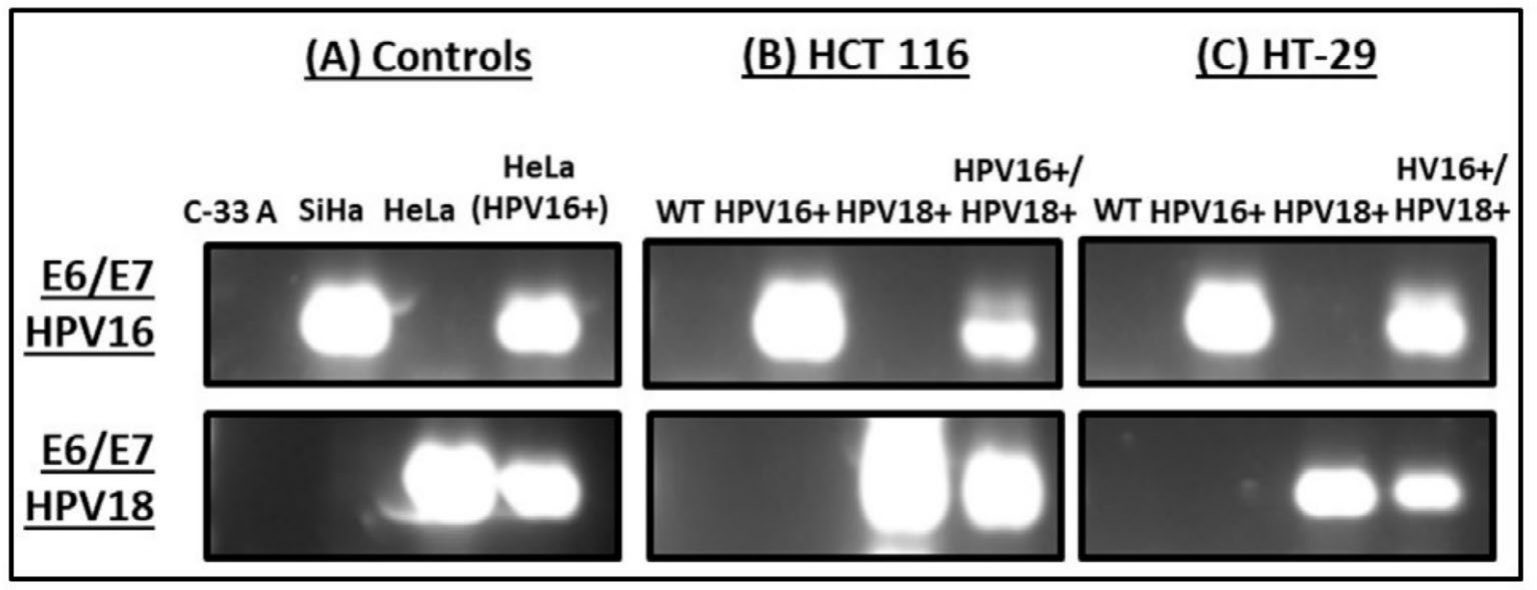
RT‐PCR analysis of HPV16+, HPV18+, and HPV16+/18+ cell lines. E6/E7 of HPV16 and HPV18 were detected at a band size of 290 and 450 base pairs respectively. (A) Cervical cancer cells, C‐33 A, SiHa, HeLa, and HeLa(HPV16+) were used as the controls for HPV‐negative, HPV16+, HPV18+, and HPV16+/18+ cells respectively. Both (B) HCT 116 and (C) HT‐29 cell lines showed the expression of E6/E7 of HPV16 or HPV18 in transfected cells and E6/E7 of HPV16 and HPV18 in co‐transfected cells.

In order to assess and compare the tumorigenic effects of single versus multiple infections with high‐risk HPVs (HPV16 and HPV18), across diverse mutational classes of CRC (KRAS and TP53 mutants), we transfected CRC cell models (HCT 116 and HT‐29) with E6/E7 of HPV16 and HPV18 alone and in combination (1:1). As a result, we obtained two transfected (HPV16+, HPV18+) and one co‐transfected (HPV16+/18+) stable cell line for each cell type. We confirmed that all stably transfected cell lines express E6/E7 of HPV16 and/or HPV18 (alone or in combination) by RT‐PCR (Figure 1). The cervical cancer cells C‐33 A, SiHa, and HeLa were used as controls for HPV‐negative, HPV16+, and HPV18+ expression respectively. HeLa cells transfected with E6/E7 of HPV16 served as a positive control for HPV16+/18+ cells.

**FIGURE 2:**
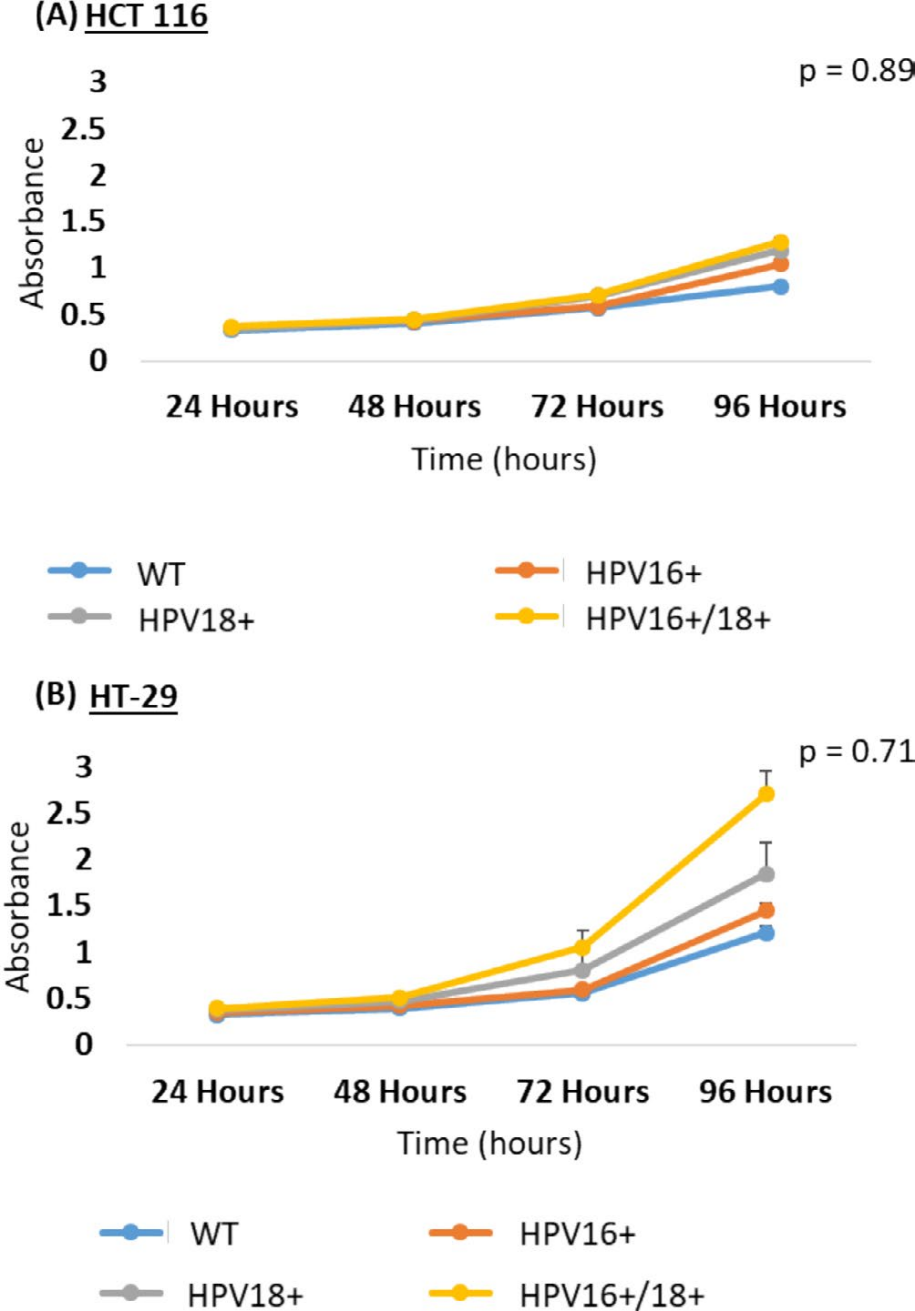
Effect of E6/E7 oncoproteins in HPV16+, HPV18+, and HPV16 + 18 co‐transfected (A) HCT 116 and (B) HT‐29 cells on cellular growth and proliferation as assessed by the CCK‐8 assay. Absorbance readings were taken at 24, 48, 72, and 96 h after commencing the assay. Data is presented in comparison to control cells. In general, a higher proliferation was observed in the HPV16+/18+ co‐transfected cells as compared to the HPV16+ or HPV18+ transfected cells.

**FIGURE 3:**
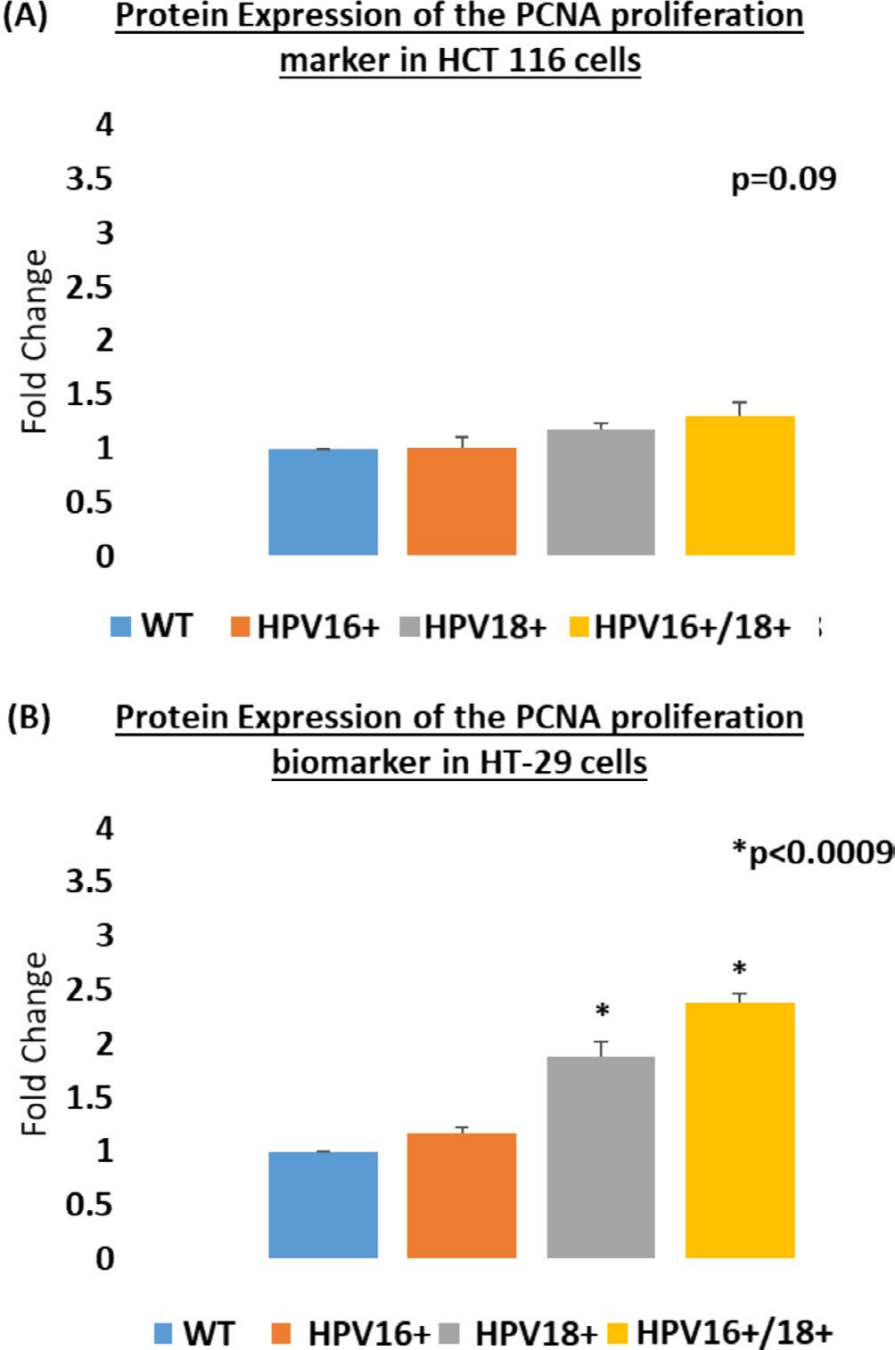
Protein expression of the cell proliferation biomarker PCNA in (A) HCT 116 and (B) HT‐29 cells transfected with HPV16 and HPV18 and co‐transfected with HPV16 + 18. PCNA expression is directly related to cell proliferation and growth. Data is presented in comparison to control cells. Significantly higher proliferation was observed in HT‐29 cells, post‐transfection with HPV (*p* = 0.0009). In general, a higher proliferation was observed in the HPV16+/18+ co‐transfected cells as compared to HPV16 or HPV18 transfected cells in both cell lines.

We initially analyzed the proliferative ability of the HPV16+, HPV18+, and HPV16+/18+ cells using the CCK‐8 formazan‐based assay. In both cell models, an increase in proliferation was observed in the order of HPV16+ < HPV18+ < HPV16+/18+ cells (Figure 2), as compared to non‐transfected controls. Among these, the highest increase in proliferation post‐transfection was observed in HT‐29 cells. However, no statistical significance was noted in these results, as determined by *p*‐values of 0.8900 and 0.7100 for HCT 116 and HT‐29 cells, respectively. Further, we also assessed the proliferative potential of transfected and co‐transfected cells via the expression of the well‐known cell proliferation marker protein, PCNA. The HPV16+/18+ (co‐transfected) cells showed a higher increase in cell proliferation as compared to HPV16+ or HPV18+ cells. However, a statistically significant increase was observed only in HT‐29 HPV16+/18+ cells (*p* = 0.0009). Similarly, HPV18+ cells showed a slightly higher cellular proliferation as compared to HPV16+ cells when compared to the non‐transfected controls in both cell models (Figure 3A,B). However, a statistically significant increase in proliferation was noted only in E6/E7 transfected HT‐29 cells (*p* = 0.0009).

Evidently, both methods assessing proliferation among E6/E7 transfected/co‐transfected cells determined that cellular proliferation was found to increase in the order of HPV16+ < HPV18+ < HPV16+/18+ cells across both cell lines, and E6/E7 of HPV18 was more potent in increasing the proliferative potential of transfected cells as compared to E6/E7 of HPV16.

**FIGURE 4:**
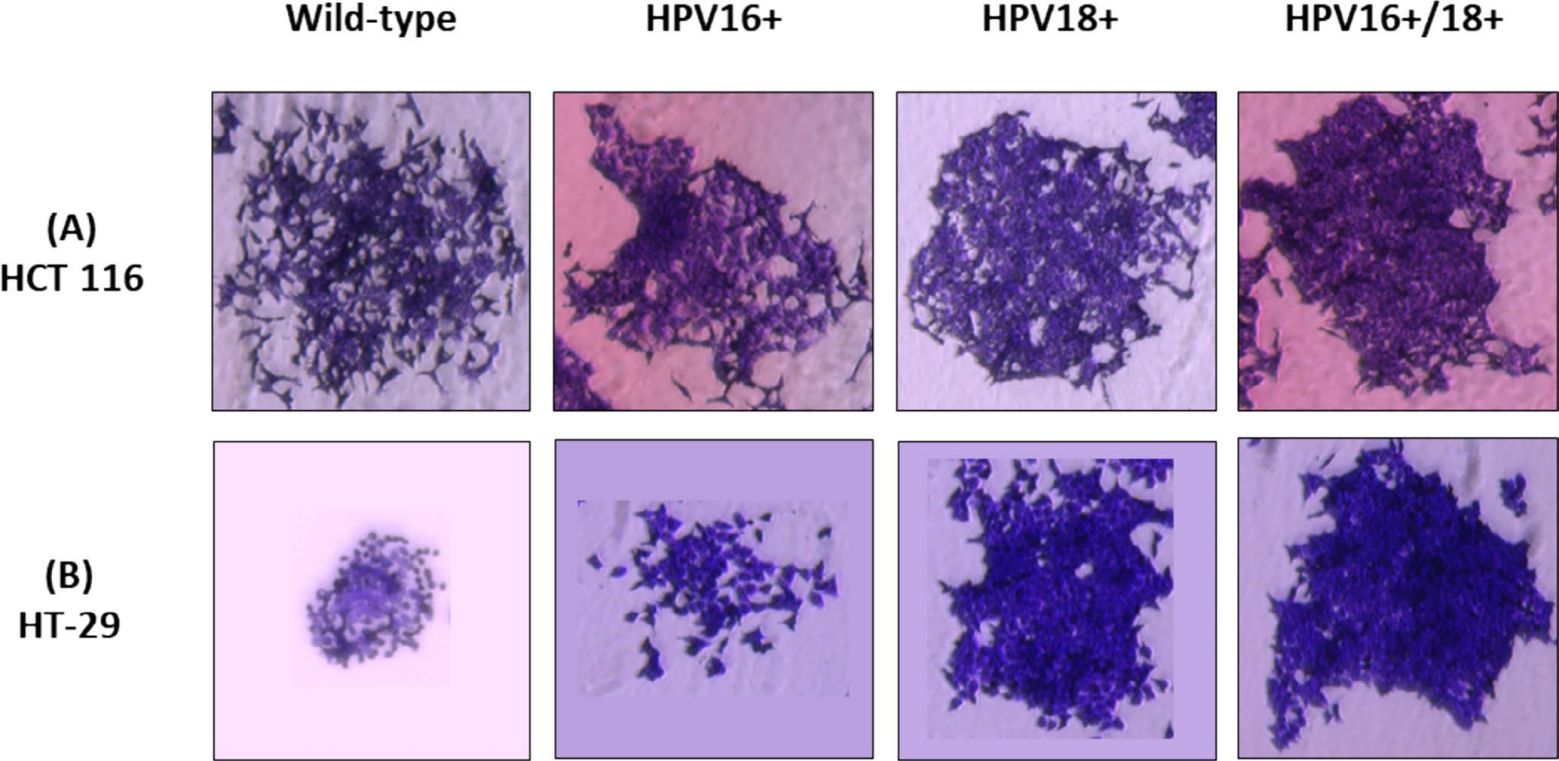
Showing a qualitative assessment and comparison of the ability of single cells to form colonies in E6/E7 transfected/co‐transfected cell lines. Single cells were suspended in a 6‐well plate (7000 cells/well) and allowed to form colonies for 11 days. Images were taken on day 11 at 4X magnification, (A) HCT 116 cell colonies were visibly denser in HPV16+/18+ cells, while only a marginal increase in the size of colonies was observed in the order of HPV16+ < HPV18+ < HPV16+/18+ cells as compared to the non‐transfected control cells. (B) A marked increase in the size and density of colonies was observed in the HT‐29 cells in the order of HPV16+ < HPV18+ < HPV16+/18+, as compared to the non‐transfected control cells.

Furthermore, we qualitatively assessed the survival/colony formation potential of single cells in order to draw a comparison between the transfected/co‐transfected and wild‐type cells. In the HT‐29 cell model, a gradual increase in the size and density of individual colonies was identified in the order of HPV16+ < HPV18+ < HPV16+/18+ cells (Figure 4B). Co‐transfected (HPV16+/18+) HT‐29 cells appeared to be dense, with cells growing as multi‐layers. On the other hand, single colonies in both transfected (HPV16+/HPV18+) and co‐transfected (HPV16+/18+) HCT 116 cells appeared to be denser compared to the non‐transfected (wild‐type) control; however, only a marginal increase in the size of colonies was observed in the order of HPV16+ < HPV18+ < HPV16+/18+ cells (Figure 4A).

**FIGURE 5:**
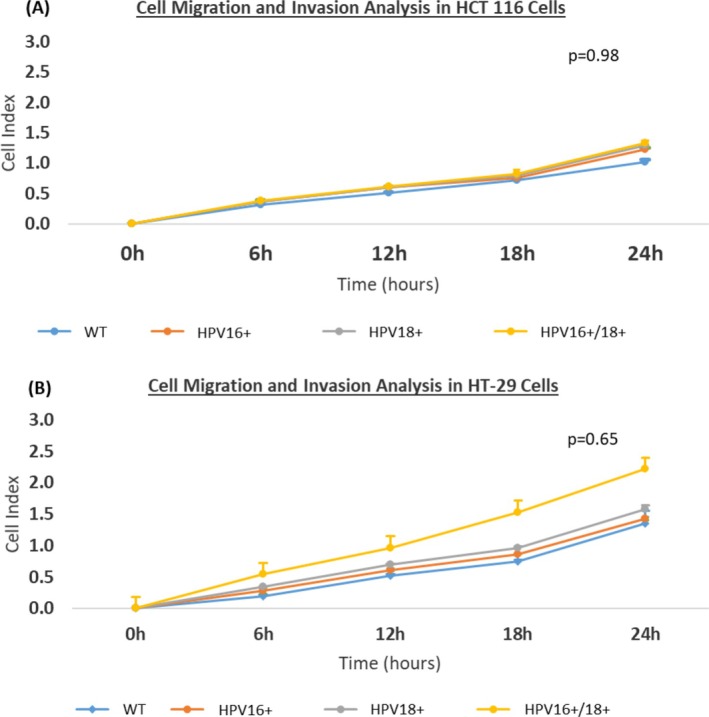
Effects of transfection with E6/E7 of HPV16 and HPV18, and co‐transfection with HPV type 16 and 18 on cell invasion and migration in (A) HCT 116 and (B) HT‐29 cells. In general, a higher cell invasion and migration were observed in HPV16+/18+ cells as compared to the HPV16+ or HPV18+ cells in both cell models.

Subsequently, we evaluated the cell migration and invasion potential of E6/E7 transfected and co‐transfected cells in comparison to the wild‐type control cells. In both cell models, an overall increase in cell invasion and migration was observed in the order of HPV16+ < HPV18+ < HPV16+/18+ cells. In the HCT 116 cell model, a fold increase of 0.20, 0.27, and 0.31 was identified in HPV16+, HPV18+, and HPV16+/18+ cells, respectively, as compared to the wild‐type control cells; however, these results failed to attain statistical significance (*p* = 0.9800) (Figure 5A). In comparison, the E6/E7 transfected and co‐transfected HT‐29 cells showed a higher increase in cell invasion and migration. Here, a fold increase of 0.10, 0.20, and 0.90 was observed in HPV16+, HPV18+, and HPV16+/18+ cells, respectively, as compared to the non‐transfected (wild‐type) control cells; however, this increase did not attain statistical significance (*p* = 0.6500) (Figure 5B).

These results indicate that the co‐transfection of E6/E7 of HPV16 and HPV18 was able to impart greater cell invasion and migration properties compared to the transfection of E6/E7 of either HPV16 or HPV18 in both CRC cell models. Also, HPV18+ cells showed an increased potential for invasion and migration compared to HPV16+ cells in both cell models. Yet, despite the statistical non‐significance in these experimental observations, such results imply that the co‐infection of high‐risk HPVs may be able to intensify the invasion and migration properties of cells compared to individuals with high‐risk HPV infections.

**FIGURE 6:**
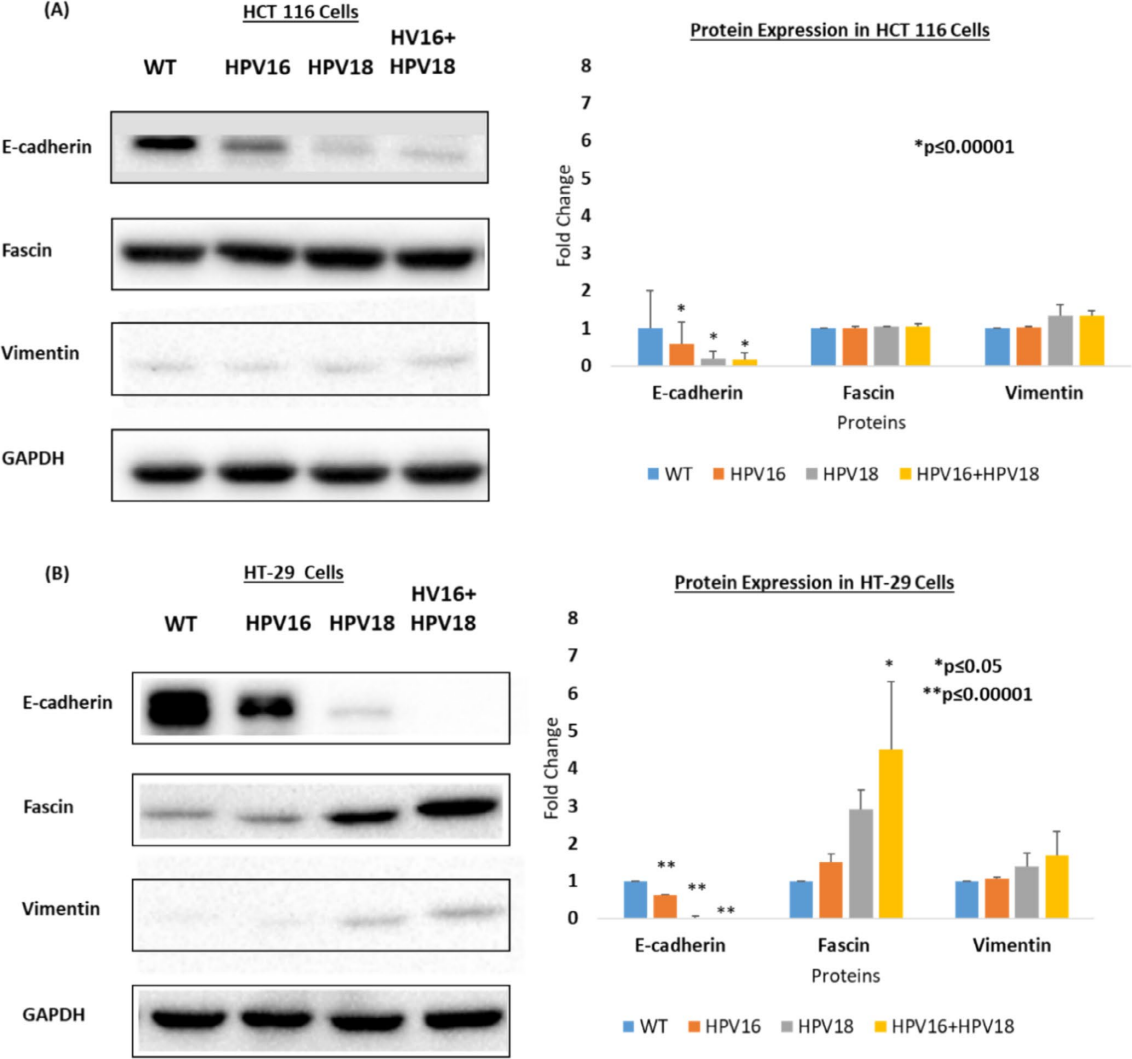
Protein expression of known biomarkers of EMT (E‐cadherin, Fascin, and Vimentin) in (A) HCT 116 and (B) HT‐29 cells transfected with E6/E7 of HPV16 and HPV18 alone and in combination. E‐cadherin was significantly downregulated in transfected/co‐transfected cells of both cell lines (*p* ≤ 0.00001). Transfected/co‐transfected HCT 116 cells showed a non‐significant upregulation of both fascin and vimentin. In the HT‐29 cells, a significant upregulation of fascin (p‐value = 0.03) was observed in HPV16+/HPV18+ cells. Data is presented in comparison to the non‐transfected control (wild‐type) cells.

Finally, we explored the expression patterns of EMT‐specific biomarkers implicated in the processes of cell migration and invasion. Here, we evaluated the expression levels of E‐cadherin, fascin, and vimentin in the transfected and co‐transfected cells in comparison to the non‐transfected control cells. Both cell models HCT 116 and HT‐29 are known to stably express E‐cadherin in the wild‐type cells. However, transfection/co‐transfection with E6/E7 greatly downregulated the expression of E‐cadherin in both cell lines. A highly significant (*p* ≤ 0.00001) reduction of E‐cadherin expression was observed in the order of HPV16+ < HPV18+ < HPV16+/18+ in both cell models. Interestingly, E6/E7 transfected/co‐transfected HT‐29 cells showed a greater downregulation of E‐cadherin as compared to E6/E7 transfected/co‐transfected HCT 116 cells. Also, HPV18+ cells showed greater downregulation of E‐cadherin as compared to HPV16+ cells in both cell lines (Figure 6A,B).

Vis‐a‐vis the expression of fascin, in general, fascin is stably expressed in wild‐type HCT 116 cells and weakly expressed in wild‐type HT‐29 cells. However, transfection/co‐transfection with E6/E7 of HPV16 and/or HPV18 led to an elevation in fascin expression in both cell lines in the order of HPV16+ < HPV18+ < HPV16+/18+ cells. In this light, a significant increase was noted only in the HT‐29 cell model (*p* = 0.0300) (Figure 6A,B).

Further, the wild‐type HCT 116 and HT‐29 cells are epithelial‐like cells; therefore, they do not express vimentin (a mesenchymal cell biomarker). However, transfection/co‐transfection with E6/E7 of HPV16 and/or HPV18 led to a marginal increase in the expression of vimentin in both cell models in the order of HPV16+ < HPV18+ < HPV16+/18+ (Figure 6A,B). Thus, the gene expression data presented here suggest a strong plausible role of high‐risk HPV infection/co‐infection in the dysregulation of EMT biomarkers, thus contributing to the progression of CRC.

## 4 Discussion

To the best of our knowledge, this is the first study aiming to illuminate and compare the oncogenic effects of single and multiple high‐risk HPV infections in cancer cells. To model these infections in CRC, we transfected and co‐transfected E6/E7 oncoproteins of HPV16 and 18 in KRAS mutant HCT 116 and TP53 mutant HT‐29 cell lines. In particular, we focused on their effect on cell proliferation, migration, invasion, and survival, which classify as critical biological processes associated with cancer progression. We also evaluated the expression patterns of EMT biomarkers like E‐cadherin [57, 58, 59, 60], fascin [61, 62, 63], and vimentin [60, 64, 65], as a major event of cancer progression. Based on the existing scientific evidence, KRAS and TP53 mutations represent nearly 52% [12, 13] and 43% [14] of the overall types of CRCs, respectively. Thus, we selected the HCT 116 and HT‐29 CRC cell lines as representatives of KRAS and TP53 mutant CRC, respectively.

### 4.1 Oncoproteins of High‐Risk HPVs Synergize in Inflicting Carcinogenic Traits

Several studies have corroborated the notion that HPV enhances cellular proliferation [73, 74, 75, 76]. Interestingly, our analysis of cellular proliferation via the CCK‐8 assay and the PCNA protein expression analysis showed a gradual increase from transfected to co‐transfected cells across both CRC cell models. In fact, a highly relevant significance (*p* = 0.0009) was noted in the PCNA expression levels in HT‐29 cells, showing an increase in the order of HPV16 < HPV18 < HPV16+/18+ cells. Similarly, our analysis also showed that HPV16+/18+ co‐transfected cells had a greater migratory and invasive potential as compared to HPV16+ and HPV18+ cells in both cell lines. These findings support the idea that the E6/E7 oncoproteins of high‐risk HPVs synergize in worsening the progression of cancer during co‐infections. These findings are also supported by recent studies in colorectal cancer patient cohorts where co‐infection with different types of high‐risk HPV, as well as other oncoviruses, was found to be a strong indicator of advanced‐stage disease [41, 69, 77].

Further, the assessment of protein expression levels of known EMT biomarkers also provided an enhanced understanding of the cellular migration and invasion capacity of E6/E7 transfected versus co‐transfected cells. The wild‐type cells of HCT 116 and HT‐29 are known to show varying expression levels of the EMT biomarkers, E‐cadherin, fascin, and vimentin, as stated by previous studies [78, 79, 80]. E‐cadherin is identified as a Type I classical cadherin that is primarily expressed on epithelial cells, where it plays a major role in cell adhesion [81]. The cadherin switch that inversely regulates the expression levels of E‐cadherin and N‐cadherin or P‐cadherin is critical for the maintenance of adheren junction [82]. It is ideally known to function as a tumor suppressor protein; hence, loss of E‐cadherin is commonly linked to EMT and metastasis [83]. Several human carcinomas are commonly linked to the decreased or aberrant expression of E‐cadherin [84, 85]. In this light, our study reported a significant decrease in E‐cadherin expression that was higher in the HPV16+/18+ cells as compared to the HPV16+ or HPV18+ cells in both cell lines. Therefore, these results strongly indicate that the E6/E7 oncoproteins of HPV16 and HPV18 indeed cooperate in downregulating EMT proteins like E‐cadherin, thereby favoring EMT and/or metastasis.

On the other hand, Fascin expression has also been associated with increased metastatic capacity in colorectal carcinogenesis [79]. In particular, Fascin has been strongly implicated in the cytoskeletal modulation of colorectal carcinomas [86, 87]. In this light, previous reports have stated that the upregulation of fascin is linked to EMT events [61, 62, 63]. In the current analysis, we observed a significant increase in the expression of fascin (*p* = 0.0300) in only the HT‐29 cell model. This increase was observed to occur in the order of HPV16 < HPV18 < HPV16+/18+ cells. As the wild‐type HT‐29 cells are known to show a weak expression of fascin, these results indeed highlight the role of high‐risk HPV in promoting cell motility and migration, particularly during co‐infections. Remarkably, another study [79] also noted that inducing fascin expression in HT‐29 cells enhanced cellular invasion, thereby mirroring our findings, where fascin upregulation occurred via transfection/co‐transfection with E6/E7 of HPV16 and/or HPV18.

In addition, vimentin is a well‐known marker of mesenchymal cells that is associated with cell invasion [88, 89, 90]. In colorectal carcinogenesis, vimentin is identified as a predictive marker for lymph node metastasis, higher tumor grade, poor prognosis, and decreased survival [91, 92]. Other reports have highlighted the roles of vimentin in cellular proliferation, migration, invasion, and particularly EMT [93, 94] in CRC and other solid cancers. Vimentin is originally expressed in trace amounts in HCT 116 and HT‐29, as reported in previous studies [95, 96]. Nevertheless, post‐transfection with E6/E7 of HPV16/HPV18, a marginal increase in the expression of vimentin was observed in both cell lines in the order of HPV16+ < HPV18+ < HPV16+/18+. The increase in vimentin expression in the co‐transfected (HPV16+/18+) cells, albeit non‐significant, raises intriguing questions regarding the mutual cooperation between the E6/E7 oncoproteins of HPV16 and HPV18 during co‐infections and their influence on EMT. Evidently, our findings strongly suggest a synergistic role of high‐risk HPV E6/E7 oncoproteins in regulating the expression levels of EMT proteins commonly associated with colorectal carcinogenesis. Taken together, these findings present significant clinical implications for CRC patients, suggesting that high‐risk HPV co‐infections may serve as a pivotal factor in accelerating cancer progression to advanced stages and heightening the risk of metastasis. This underscores the importance of developing targeted preventive and therapeutic strategies to address this issue.

### 4.2 Oncoproteins of HPV16 and HPV18 Differ in Their Oncogenic Potential

There is currently limited evidence comparing the oncogenic potential of the two most frequently observed HPVs (HPV16 and HPV18) worldwide. Herein, we present a comparative assessment of the effects of the E6/E7 oncoproteins of HPV16 and HPV18 on cell proliferation, migration, invasion, and survival, as well as on the expression patterns of EMT proteins. The results obtained from the CCK‐8‐based proliferation analysis indicated that E6/E7 of HPV18 induced a higher proliferative effect in both HCT 116 and HT‐29 cell lines, as compared to E6/E7 of HPV16. Similarly, the overall expression of the PCNA proliferation marker was found to be higher in HPV18+ cells as compared to HPV16+ cells in both cell models. In addition, HPV18+ cells identified with a marginally higher induction in cellular invasion and migration in comparison to HPV16+ cells in both HCT 116 and HT‐29 cells. Likewise, deregulation of the EMT biomarkers E‐cadherin, fascin, and vimentin in both CRC cell models was more pronounced in HPV18+ cells as compared to HPV16+ cells. Hence, these findings suggest that HPV18 might exhibit greater efficacy in promoting and sustaining malignant characteristics in CRC cell models, as compared to HPV16. These results are also in agreement with another study on cervical cells [97] which demonstrated that only HPV18‐immortalized cells were able to exhibit anchorage‐independent growth, while HPV16‐immortalized cells failed to compare in similar oncogenic characteristics. Yet, such effects may only be limited to in vitro models; therefore, further analysis in in vivo disease models and patients with high‐risk HPV‐associated CRC cells may be required to determine the certainty of these findings.

### 4.3 Variability in Oncogenic Effects of High‐Risk HPVs Based on CRC Mutational Status

Our data from the cell proliferation analyses showed a marked increase in the proliferation of HPV16+, HPV18+, and HPV16+/18+ cells in the TP53 mutant HT‐29 cells as compared to the KRAS mutant HCT 116 cells. Similarly, a much higher increase in cell invasion and migration was noted in HT‐29 cells as compared to HCT 116 cells post‐transfection with E6/E7 of HPV16 and/or HPV18. In addition, a higher downregulation of E‐cadherin and upregulation in fascin and vimentin expression was noted in the HPV16+, HPV18+, and HPV16+/18+ cells of the HT‐29 cell model as compared to the matched cells in the HCT 116 cell model. Therefore, these results strongly indicate that the E6/E7 oncoproteins of high‐risk HPVs may be more effective in modulating cancer gene expression in TP53 mutant CRC as compared to KRAS mutant CRC. This may be due to the inherent attribute of the E6 and E7 oncoproteins to repress the functioning of the TP53 gene. For example, E6 is known to bind to the p53 protein and promote its degradation [98]. Therefore, HPV‐associated cancers are found to express low or aberrant levels of p53 [98, 99]. This is accompanied by further downstream oncogenic deregulation by E7 which operates in a low‐p53 environment [100]. Therefore, high‐risk HPVs may be widely potent in promoting malignant phenotypes in TP53 mutant CRC where p53 levels are scarce.

Accordingly, this study emerges as a pioneer in assessing the effects of the E6/E7 oncoproteins of high‐risk HPVs in the diverse mutational classes of CRC. Therefore, our results may be used as a stepping‐stone to future studies directed at analyzing the impact of high‐risk HPVs in CRCs of heterogeneous mutational backgrounds. In addition, these findings underscore the profound impact of mutational heterogeneity in HPV‐associated cancers like CRC, reinforcing the critical need for personalized therapeutic strategies that are precisely tailored to the unique genetic makeup of each tumor.

## 5 Conclusion

Our investigation established a robust model to compare and contrast the effects of single and multiple infections of high‐risk HPVs in CRC. In this light, our study unveiled the synergistic interplay between the E6/E7 oncoproteins of the highly prevalent high‐risk HPVs (HPV16 and HPV18) in regulating cell proliferation, migration, invasion, survival, and the expression of EMT‐related proteins, thus underscoring their pivotal roles during co‐infections. Our investigation also evidences the contrasting activity of the E6/E7 oncoproteins of high‐risk HPVs in two distinct mutational cell models of CRC. These results indicate that E6/E7 may modulate different sets of oncogenes and signaling pathways in KRAS and TP53 mutational models of CRC, thus amplifying the need for personalized therapies for diseases like CRC.

While coinfection with different types of high‐risk HPVs has been demonstrated previously to be linked with advanced‐stage colorectal cancer, further in vivo analysis, preferably in animal models, is essential to validate our findings. Additionally, other in vitro models including normal epithelial colorectal cells for high‐risk HPV coinfections would be valuable to uncover additional molecular and cellular aspects, such as key signaling molecules and pathways activated during coinfections. This would provide a more comprehensive context for our study's findings.

## Author Contributions

Conceptualization: Ala‐Eddin Al Moustafa. Writing – original draft preparation: Queenie Fernandes. Sample Processing: Queenie Fernandes, Varghese Philipose Inchakalody, Sarra Mestiri, Takwa Bedhiafi, Shereena Hydrose, Sara S. Bashraheel. Statistical Analysis: Queenie Fernandes, Varghese Philipose Inchakalody. Review and editing: Maysaloun Merhi, Said Dermime, and Ala‐Eddin Al Moustafa. All authors have read and agreed to the published version of the manuscript.

## Conflicts of Interest

The authors declare no conflicts of interest.

## Data Availability

Data available on request from the authors.
